# Intermittent low-intensity and moderate-intensity exercise effects on cognition in community-dwelling older adults: a pilot study exploring biological mechanisms

**DOI:** 10.3389/fnagi.2024.1432909

**Published:** 2024-10-17

**Authors:** Swathi Gujral, Judy L. Cameron, Kayla Conaty, Sumer Ziady, Amrita Sahu, John M. Jakicic, Renee J. Rogers, Caterina Rosano, Abbe N. Vallejo, Kirk I. Erickson, Tamer S. Ibrahim, Howards Aizenstein, Charles F. Reynolds, Meryl A. Butters

**Affiliations:** ^1^Department of Psychiatry, University of Pittsburgh School of Medicine, Pittsburgh, PA, United States; ^2^Western Psychiatric Hospital, University of Pittsburgh Medical Center, Pittsburgh, PA, United States; ^3^Department of Physical Medicine and Rehabilitation, University of Pittsburgh School of Medicine, Pittsburgh, PA, United States; ^4^Department of Environmental and Occupational Health, University of Pittsburgh Graduate School of Public Health, Pittsburgh, PA, United States; ^5^Department of Internal Medicine, Division of Physical Activity and Weight Management, University of Kansas Medical Center, Kansas, KS, United States; ^6^Department of Epidemiology, University of Pittsburgh Graduate School of Public Health, Pittsburgh, PA, United States; ^7^Department of Neuroscience, AdventHealth Research Institute, Orlando, FL, United States; ^8^Department of Psychology, University of Pittsburgh, Pittsburgh, PA, United States; ^9^Department of Bioengineering, University of Pittsburgh, Pittsburgh, PA, United States

**Keywords:** exercise, cognition, aging, brain, inflammation

## Abstract

**Background/objective:**

To examine the cognitive benefits of 6 months of prescribed intermittent exercise (10-min bouts totaling 150 weekly minutes) in community-dwelling older adults, comparing effects of low-intensity movement (LIM) and moderate-intensity aerobic exercise (aerobic exercise; AE) training; and exploring biological mechanisms of exercise-related cognitive improvement.

**Method:**

Twenty-five adults (>60 years old) participated in a 6-month controlled trial and were randomized into LIM or AE intermittent training. Cognition was assessed using a neuropsychological test battery including the Repeatable Battery for the Assessment of Neuropsychological Status (RBANS), California Verbal Learning Test, 2nd Edition (CVLT-II), and Delis-Kaplan Executive Function System (D-KEFS). Neuroimaging measures were collected using a 7 T human MRI scanner. Serologic neurotrophic and inflammatory factors were analyzed using Luminex multiplex assays [brain derived neurotrophic factor (BDNF); vascular endothelial growth factor (VEGF)]; interleukin-6 (IL-6), C-reactive protein (CRP), plasminogen activator inhibitor (PAI-1).

**Results:**

LIM and AE intermittent training had dissociable effects on cognition, with LIM resulting in improved learning and memory and AE resulting in improved executive functioning. Intervention groups differed on change in cognitive performance on CVLT-II learning and D-KEFS trail making test. Increase in right dorsolateral prefrontal cortex (DLPFC) surface area was linked to executive improvement (i.e., phonemic fluency) regardless of intervention group. A decline in circulating PAI-1 was linked to learning and memory improvement in response to LIM over 6 months.

**Conclusion:**

Moderate-intensity AE and LIM intermittent training likely have distinct cognitive benefits, though low-intensity activity is often included as a control group in exercise trials in aging.

## Introduction

Physical activity (PA) is a promising and cost-effective behavioral intervention for improving cognitive and brain health among older adults. Exercise interventions, in as short as 6-months have been shown to have meaningful benefits for cognitive function and brain health among cognitively unimpaired and mildly impaired older adults (Angevaren et al., 2008; Erickson et al., 2019; Erickson et al., 2011; Suzuki et al., 2013; Zheng et al., 2016). Meta-analyses of RCTs report favorable small-to-moderate effects (range: 0.10–0.70) of AE on cognitive performance in cognitively unimpaired older adults, depending on the duration of AE and outcome (Kramer and Colcombe, 2018; Zheng et al., 2016). Emerging evidence in those with Mild Cognitive Impairment (MCI) similarly suggests a moderate positive effect of AE on cognition (range: 0.30–0.70) (Biazus-Sehn et al., 2020). Neuroimaging studies of exercise training in aging, namely aerobic exercise, suggest exercise influences structural brain integrity and functional dynamics in a regionally specific pattern, with the most consistent effects observed in the prefrontal cortex and hippocampus (Wilckens et al., 2021; Zheng et al., 2019). Systemic adaptations to exercise training results in the release of molecular factors into circulation from numerous organ systems (i.e., skeletal muscle, adipose tissue, liver), which ultimately enhance the function of distal organs, such as the brain, by promoting vascularization, neurogenesis, and synaptogenesis (Chow et al., 2022; Gubert and Hannan, 2021). Key potential molecular and cellular mechanisms for the effects of *aerobic* exercise on cognition and brain health include cardiovascular effects (e.g., cardiorespiratory fitness, increase in factors promoting angiogenesis); anti-inflammatory effects (i.e., reduction in systemic inflammatory signaling), and neurotrophic effects (i.e., increase in brain-derived neurotrophic factor (BDNF) in hippocampus) (Stillman et al., 2016). In sum, there is a convincing literature supporting the cognitive and brain health benefits of aerobic exercise in older adults and support for several likely mechanisms that explain these benefits through pre-clinical and translational studies.

Though low-intensity exercise modalities such as stretching and other low-intensity forms of movement are frequently included as a control condition across many exercise trials, little work has focused on describing any distinct biological and cognitive changes that occur with low-intensity movement vs. moderate-intensity aerobic exercise. The literature also fails to acknowledge that beyond being at a lower-intensity, prolonged exercise programs involving stretching and other forms of low intensity movement are characteristically distinct from moderate-intensity aerobic exercise. For instance, stretching routines often have components of interoceptive sensitivity, balance and flexibility, and the novelty of these exercise routines may require a greater amount of sensorimotor learning relative to typical modes of aerobic exercise (e.g., brisk walking) (Price and Hooven, 2018). The cognitive benefits and the biological mechanisms underlying potential cognitive benefits of low-intensity stretching and movement remain poorly understood; however, an emerging literature suggests mindful low-intensity movement practices (e.g., yoga, Tai Chi) may improve cognitive and brain health via improving HPA-axis regulation, an increase in attentional capacity, and an increase in interoceptive awareness (Chan et al., 2019; Muehsam et al., 2017; Tao et al., 2019; Wu et al., 2019; Zou et al., 2019). Whether these same benefits may be observed with general stretching and low-intensity movement in older adults in unknown.

Over the past two decades, there has been much discussion and controversy in the literature regarding whether the benefits of PA depend on minimal duration of PA bouts (i.e., 10-min bouts) (Chang et al., 2015; Loprinzi et al., 2018; Sanders et al., 2019). The 2018 National Physical Activity Guidelines Scientific Report concluded that health-related benefits of PA occur regardless of bout duration (Jakicic et al., 2019; Piercy et al., 2018). Yet, evidence suggests PA accrued in short bouts (e.g., 10-min) rather than longer PA sessions (e.g., 50-min) may promote PA adherence among previously sedentary adults (Jakicic et al., 1995). The present 6-month pilot exercise trial targeting cognition in community-dwelling older adults involved a prescription of intermittent PA (three 10-min bouts of PA per day, totaling 150 min per week) for participants randomized to both the LIM (*n* = 10) condition and moderate-intensity AE (*n* = 15) condition. This pilot study aimed to examine the extent to which 6 months of *brief intermittent bouts of* moderate-intensity AE relative to LIM results in similar cognitive and brain health changes as shown in previous studies involving 6 months of AE and LIM accrued in *longer, less frequent bouts* (e.g., 50-min sessions). We predicted that intermittent moderate-intensity AE would result in (a) greater improvement in memory and executive functioning and (b) greater morphological changes in the dorsolateral prefrontal cortex (DLPFC) and hippocampus, based on previous evidence of moderate-intensity AE-related brain changes among older adults. We further explored whether changes in select circulating biomarkers implicated in brain aging [i.e., insulin growth factor (IGF-1), plasminogen activator inhibitor (PAI-1), vascular endothelial growth factor (VEGF), and brain derived neurotrophic factor (BDNF)] may explain the cognitive benefits of intermittent moderate-intensity AE vs. LIM.

## Methods

### Participants

Participants included 25 inactive, cognitively healthy, community-dwelling older adults 65 years old and older. An inactive lifestyle was defined by engaging in less than 60 min of weekly moderate-to-vigorous physical activity. Primary Care Provider clearance was required for study participation to ensure exercise safety and participants had to be cleared to undergo a MRI. Exclusionary criteria included mobility limitations (e.g., use of an assistive device for walking), congestive heart failure, myocardial infarction in the past 12 months, history of stroke, uncontrolled hypertension, or diagnosis of dementia or cognitive performance in the dementia range on a cognitive screening test [Modified Mini Mental State Exam (3MS) score < 85], and heavy current alcohol use (>1 alcoholic drink 5+ days per week).

### Assessments

#### Cognitive function

A comprehensive neuropsychological test battery (2.5 h) with national age-based norms was administered to participants at baseline and after completion of the 24-week intervention. The cognitive assessment battery was comprehensive and emphasized evaluation of cognitive domains known to be susceptible to age-related decline and sensitive to exercise training (i.e., attention, information processing speed, executive functioning, learning and memory). A word reading and decoding measure, the Wide Range Achievement Test (4th edition) Reading Subtest (Wilkinson and Robertson, 2006) was used to estimate premorbid verbal intellectual ability. Overall cognitive function was assessed with the Repeatable Battery for the Assessment of Neuropsychological Status (RBANS) (Randolph, 1998). Basic attention and working memory were assessed more in-depth using the Wechsler Adult Intelligence Scale, 4th Edition (WAIS-IV) Digit Span subtest (Wechsler, 2008). Learning and memory were assessed in more detail using the California Verbal Learning Testing, 2nd Edition (CVLT-II) (Delis et al., 2000). Executive function was assessed using the Delis-Kaplan Executive Function System (D-KEFS) Trail Making Test (speeded set-shifting) and D-KEFS Color Word Interference Test (inhibition) (Delis et al., 2001), and verbal fluency measures (Controlled Oral Word Association Test and animal naming test) (Benton et al., 1983). Cognitive test forms were counterbalanced across participants and alternate forms were used for the post-intervention cognitive assessment for the RBANS and CVLT-II to minimize possible practice effects.

#### MR image acquisition

Neuroimaging data were collected on a Siemens 7 T Siemens Magnetom scanner with the 1st generation Tic Tac Toe radiofrequency (RF) coil system at the University of Pittsburgh. The T1-weighted 3D MPRAGE sequence (256 slices (0.5 Iso.); TR 3000; TE 2.56) was used for volumetric analyses of the total hippocampus and hippocampal subfields (Krishnamurthy et al., 2019; Santini et al., 2021; Santini et al., 2018). Cortical reconstruction and volumetric segmentation was performed with the FreeSurfer image analysis suite, which is documented and freely available for download online^1^ (Reuter et al., 2012). FreeSurfer’s hippocampal subfield segmentation protocol (Iglesias et al., 2015) was used to perform segmentation from the MPRAGE image. Per FreeSurfer’s protocol, the following hippocampal subfields were extracted from each hemisphere: dentate gyrus (DG)/Cornu Ammonis (CA)-4, CA-2/3, CA-1, pre-subiculum/subiculum, and the molecular layer (of the CA and subiculum subfields, combined). DG/CA-4 were combined because the CA-4 subfield lies within the dentate gyrus in FreeSurfer’s hippocampal subfield atlas (Iglesias et al., 2015). The dorsolateral prefrontal cortex was selected as the sole region of interest for exploratory analyses examining associations between change in brain morphometry (volume, cortical thickness, surface area) and improvement in executive functioning.

#### Blood biomarkers

Fasting blood samples (6 Tbsp) were obtained from all participants at baseline and post-intervention by a trained nurse or phlebotomist. Participants were requested to refrain from any moderate-to-vigorous physical activity for 12 h prior to blood data collection. Blood was then processed and stored at −80c by another trained staff member. For our exploratory analyses aimed to identify blood biomarker targets of intermittent AE vs. LIM that may mediate cognitive changes of intermittent AE, blood serum was analyzed for protein biomarkers related to immune function and brain aging using multiplex protein array that assesses an array of molecules and protein expressions that are thought to be indicative of brain aging concerns [i.e., brain derived neurotrophic factor (BDNF); vascular endothelial growth factor (VEGF), interleukin 6 (IL-6), C-reactive protein (CRP), plasminogen activator inhibitor (PAI-1)].

#### Intervention procedures

Participants were randomized to AE or LIM intermittent training interventions for 24 weeks. These interventions were designed based on theoretical frameworks, specifically the Social Cognitive Theory (Bandura, 1986), Problem Solving Theory (Perri et al., 2001), Relapse Prevention (Marlatt and Gordon, 1985), and Stages of Motivational Readiness for Change (Prochaska et al., 1994). Strategies employed in these interventions included self-monitoring, goal setting, problem solving, mastery skills, social support, and relapse prevention.

Participants in both intervention groups were provided with a physical activity program aimed at reaching a total of 150 min per week. They were instructed to engage in physical activity sessions 5 days per week, starting with one to two prescribed 10-min daily sessions and gradually increasing to three prescribed 10-min sessions per day; however, a participant could self-select to perform a session for longer than 10 min to facilitate engagement in the prescribed dose of physical activity. Both interventions included a combination of in-person and home-based physical activity sessions (see below) and participants received regular telephone support from study staff (as specified below) to encourage adherence to the prescribed physical activity regimen. Participants were provided with electronic tablets containing instructional videos to facilitate their activity sessions. Participants were requested to monitor and record completion of their activity sessions that were consistent with their prescription. Throughout the duration of the first 2 months, participants attended one weekly in-person session lasting approximately 60 min that included a supervised exercise component and also discussion about behavioral strategies to facilitate ongoing engagement in the prescribed physical activity. Subsequently, during the third and fourth month, this frequency decreased to bi-monthly in-person supervised sessions along with two brief 10-min telephone calls per month. As the intervention progressed into the fifth and sixth month, participants attended one in-person supervised session per month, supplemented by one brief 10-min telephone call monthly. These sessions involved supervised physical activity closely resembling the prescribed physical activity, as well as interactive discussions among participants and staff on strategies to achieve the prescribed physical activity goals.

##### Low-intensity movement (LIM) intervention

Physical activity sessions in this group were prescribed at low-intensity chair-based activities that included full body stretching, joint range of motion, posture and body alignment, and relaxation. These activities were prescribed to be performed in a slow and controlled manner that were accompanied by appropriate breathing techniques to match these activities. Safety and body awareness cues were incorporated into each of the movements to assist participants in understanding what they should be feeling and how their body should be moving. To facilitate the LIM intervention, videos produced by the study were made available on a tablet provided to each participant for home use. These same videos were used during any supervised onsite activity session.

##### Moderate-intensity aerobic exercise (AE) intervention

Physical activity sessions in this group were prescribed at a moderate-intensity, which would be equivalent to the intensity of taking a brisk self-paced walk. Thus, the primary form of physical activity that was recommended for AE was self-paced brisk walking; however, other similar forms of aerobic activity could be self-selected by the participant. Similar to the LIM intervention, videos of aerobic activity produced by the study were made available on a tablet provided to each participant for home use. These same videos were used during any supervised onsite activity sessions. These activities in the videos were performed in a rhythmic manner where participants were encouraged to follow the pace of the instructor for safety, with modifications provided to match skill and fitness level. Form and breathing cues were provided for safety.

#### Statistical analysis

Intervention group differences in key demographic characteristics (age, sex, race, years of education, estimated premorbid IQ) were examined using independent samples t-tests for continuous variables and Fisher’s Exact Chi-Square tests for categorical variables. Given the small sample size, Wilcoxon rank-sum test/Mann Whitney U nonparametric tests were used to evaluate intervention group differences in percent change in performance on cognitive measures over 24 weeks. Sensitivity analyses for change scores with a normal distribution included linear regression models predicting percent change in cognitive performance, examining effects of intervention group and adjusting for any demographic variables that differed between intervention groups at baseline. Paired samples t-tests were used to explore cognitive changes within each intervention group. For brain morphological outcomes, non-parametric Spearman’s rho correlations were used to examine associations of cognitive improvement on tests showing AE- or LIM-related improvement with percent change in structural neuroimaging markers related to the respective cognitive domain of improvement (e.g., hippocampal subfield volumes for learning and memory improvement; dorsolateral prefrontal cortex volume/thickness/surface area for executive functioning improvement).

Exploratory analyses were conducted to probe circulating biomarkers of cognitive improvement related to intermittent moderate-intensity AE relative to LIM. Given the small sample size, Wilcoxon rank-sum test/Mann Whitney U nonparametric tests were used to evaluate intervention group differences in percent change in serologic levels of neurotrophic (BDNF, VEGF) and inflammatory factors (IL-6, CRP, PAI) sensitive to exercise and implicated in cognitive aging. Jamovi Version 2.3.28 was used to conduct data analyses.

## Results

### Baseline profile of study participants

**Figure 1:**
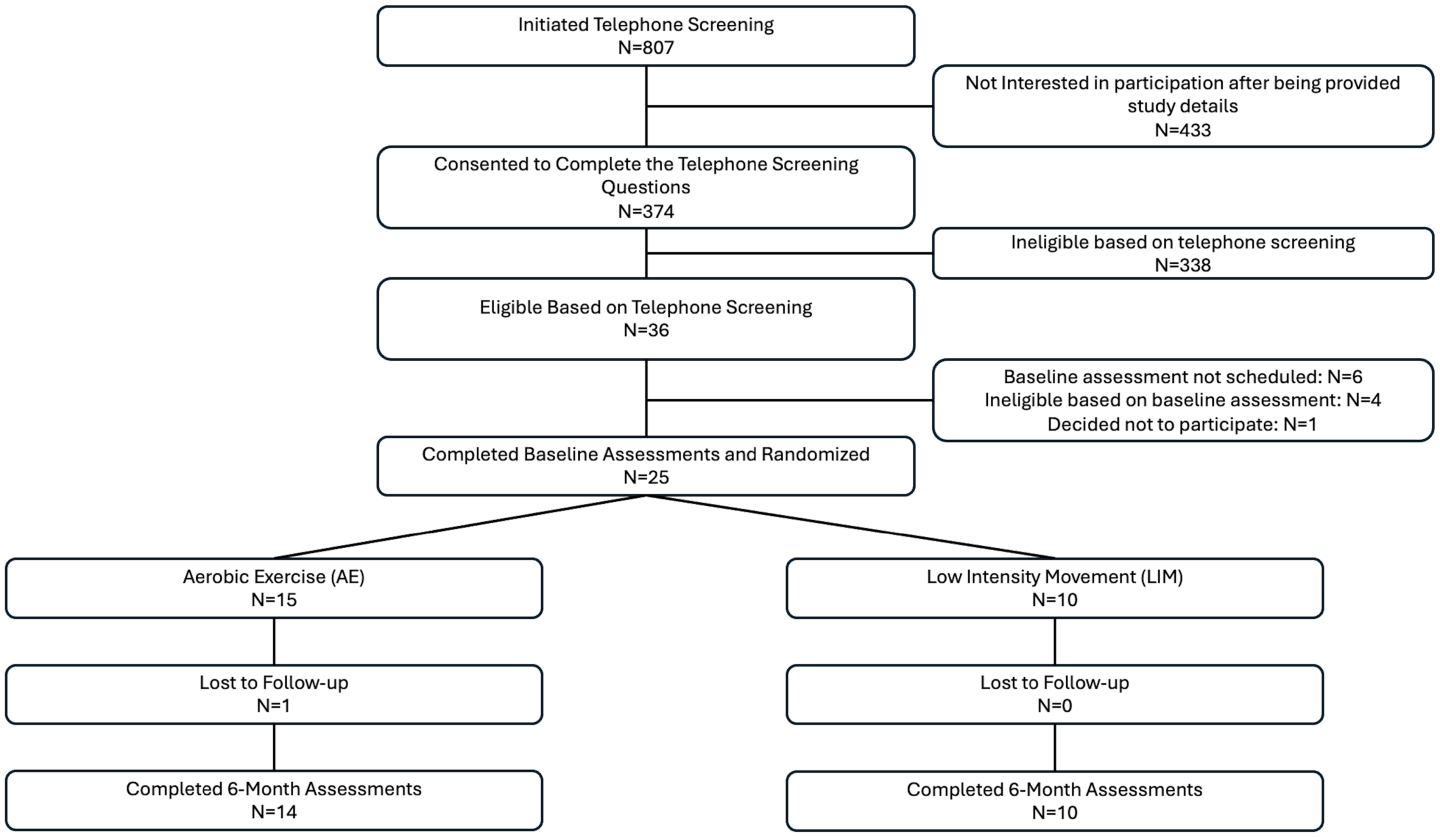
Consort diagram of study enrollment.

Details regarding study enrollment can be found in the consort diagram (Figure 1). Twenty-five participants completed this pilot intervention study, including 10 participants in LIM and 15 participants in AE. One participant did not complete all of the post-intervention assessments and was thus excluded from the analyses (*n* = 14 in aerobic exercise group). There were no group differences in demographics, neuroimaging or cognitive measures at baseline (*p* > 0.20 for all). However, intervention group differences in WRAT-4 Reading Subtest at baseline were significant, with the LIM group showing higher scores (*p* = 0.04) (see Table 1). Sensitivity analyses adjusted for the WRAT-Reading score, which was used as an estimate of premorbid IQ.

**Table 1:** Baseline demographics and clinical variables.

Variable, mean (SD) or *N* (%)	LIM (*n* = 10)	AE (*n* = 15)	*p*-value
Age (years)	69.0 (8.0)	71.0 (7.0)	0.29
Female Sex (*N*, %)	9 (90%)	11 (79.0%)	0.62
White Race (*N*, %)	9 (90%)	12 (86.0%)	0.99
Education (yrs)	15.5 (6.0)	16.0 (6.0)	0.84
PHQ-9 Total (RS)	1.7 (1.49)	1.8 (2.2)	
3MS Total (RS)	97 (2.3)	96 (3.9)	
WRAT4 Reading (SS)	115.3 (13.4)	104.5 (11.1)	

Fishers exact chi-square tests and Wilcoxon rank sum tests of medians were used to compare categorical and continuous tests, respectively; RS, Raw Score; LIM, Low-intensity movement; AE, moderate-intensity aerobic exercise; PHQ-9, Patient Health Questionnaire, 9-item Version; 3MS, Modified Mini-Mental State Exam; WRAT4, Wide Range Achievement Test, 4th edition *p ≤ 0.05; **p ≤ 0.01; ***p ≤ 0.001.

### Exercise intervention adherence

Data were examined using independent t-tests to determine if there were differences between LIM and AE for the number of activity sessions performed across the intervention period. As shows in Table 2, there were no differences between LIM and AE for the number of activity sessions completed that used the videos, in-person sessions, non-video or non-supervised sessions, or total activity sessions.

**Table 2:** Physical activity sessions completed for the intervention conditions (mean ± standard deviation).

Physical activity sessions	Low-intensity movement (LIM) (*n* = 10)	Aerobic exercise (AE) (*n* = 14)	*p*-value^*^
In-person sessions	252.7 ± 82.9	184.7 ± 84.7	0.063
video sessions	22.7 ± 3.9	23.0 ± 3.2	0.839
Non-supervised and non-video sessions	36.3 ± 50.4	62.6 ± 47.6	0.205
Total sessions	272.6 ± 131.1	270.3 ± 56.5	0.954

* *p-value for the difference between LIM and AE.*

### Exercise intervention effects on cognition

**Figure 2:**
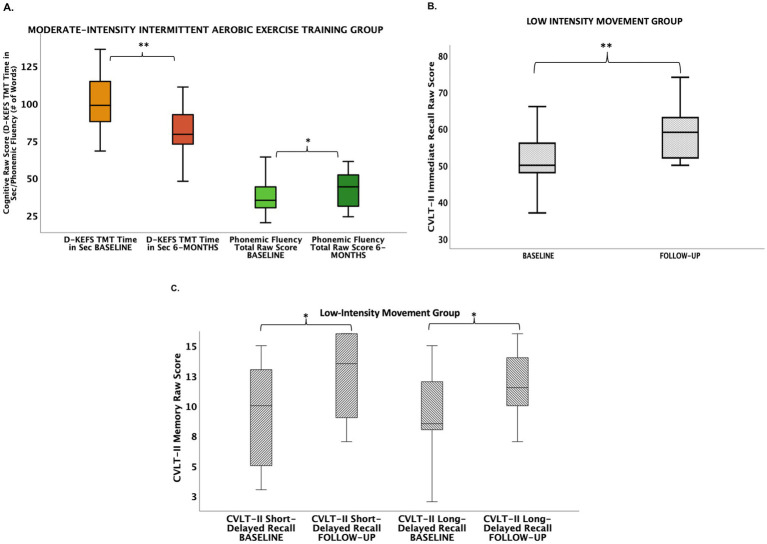
Cognitive improvements in aerobic exercise (A) and low-intensity movement intervention groups (B,C). (A) Aerobic exercise group showed improvements from baseline to 6 months in executive functions (i.e., set-shifting, phonemic fluency). (B) Low-intensity movement showed improvement from baseline to 6 months in verbal learning. (C) Low-intensity movement group showed improvement from baseline to 6 months in short-delayed verbal memory recall and long-delayed verbal memory recall. CVLT-II, California Verbal Learning Test, 2nd edition; D-KEFS TMT, Delis-Kaplan Executive Function System Trail Making Test.

Within group analyses indicated participants in both intervention groups showed cognitive improvement over 6 months. Group differences in percent change in cognitive performance scores were statistically significant for verbal learning and set-shifting (an aspect of executive functioning) [California Verbal Learning Test (CVLT-II) Immediate Recall Raw Score: LIM > AE, Table 3; Delis-Kaplan Executive Function System (D-KEFS) Trail Making Test Time in Seconds Raw Score: AE > LIM, Table 3; Figure 2]. Exploratory within-group analyses indicated the LIM group showed improvement in learning and memory (CVLT-II Immediate Recall, Short-Delayed Recall, Long-Delayed Recall), while the AE group showed improvement in executive functioning (D-KEFS Trail Making Test, Letter Fluency) after 6 months (Figure 2).

**Table 3:** Intervention effects on cognition.

Variable, median (IQR) or *N* (%)	LIM baseline (*n* = 10)	LIM post-intervention (*n* = 10)	*p*-value^1^	AE baseline (*n* = 14)	AE post-intervention (*n* = 14)	*p*-value^2^	*p*-value^3^
CVLT-II Immediate Recall RS	50.9 (8.1)	58.6 (7.4)	0.01**	47.5 (8.6)	49.2 (13.2)	0.41	0.05*
CVLT-II Short delay recall RS	9.5 (4.3)	12.8 (3.6)	0.02*	9.6 (3.2)	10.7 (4.4)	0.23	0.34
CVLT-II Long delay recall RS	9.3 (4.0)	11.9 (3.0)	0.01**	9.7 (3.2)	10.6 (4.2)	0.21	0.29
CVLT-II Retention (%)	124.7 (104.6)	95.5 (18.4)	0.39	106.1 (31.8)	101.6 (22.7)	0.64	0.84
CVLT-II Recognition Discriminability	2.9 (0.8)	3.2 (0.6)	0.30	2.8 (1.0)	2.9 (1.1)	0.71	0.93
D-KEFS Trail Making Test RS (sec)	98.6 (22.9)	109.4 (48.8)	0.40	99.8 (21.1)	80.3 (19.6)	0.002**	0.03*
D-KEFS Trail Making Test difference (scaled Score)	9.1 (2.0)	9.3 (3.4)	0.78	9.4 (1.6)	11.3 (1.8)	0.001***	0.11
COWAT Phonemic Fluency RS	46.7 (7.1)	48.5 (9.1)	0.64	37.9 (12.1)	42.2 (11.7)	0.02*	0.54

1 *p-value derived from paired t-test and answers the question: are post-intervention scores significantly different from baseline scores for LIM.*

2 *p-value derived from paired t-test and answers the question: are post-intervention scores significantly different from baseline scores for AE.*

3 *p-value used to test the difference between LIM and AE in percentage change of cognitive score from baseline to post-intervention. Wilcoxon-rank sum test used in place of t-test because of small sample size.*

RS, Raw Score; LIM, Low-intensity movement; AE, Moderate-intensity aerobic exercise; IQR, interquartile range; CVLT-II, California Verbal Learning Test, 2nd edition; D-KEFS, Delis-Kaplan Executive Function System; COWAT, Controlled Oral Word Association Test. Bold values indicate *p* values ≤ 0.05; *p ≤ 0.05; **p ≤ 0.01; ***p ≤ 0.001.

### Sensitivity analyses

Given baseline intervention group differences in the WRAT-4 Reading score (LIM > AE), which is a commonly used estimate of premorbid intellectual ability among older adults, sensitivity analyses included linear regression models adjusting for WRAT-4 Reading score. The WRAT-4 Reading subtest estimates an individual’s *crystalized* intelligence, which does not significantly decline with age. Intervention group differences in D-KEFS Trail Making Test performance (AE > LIM) remained significant after adjustment for WRAT-4 Reading Score (*B* = −31.59, SE = 11.85, *t* = −2.67, *p* = 0.01, *R*^2^ = 0.26). Intervention group differences in CVLT-II Learning performance (LIM > AE) became non-significant after adjusting for WRAT-4 Reading Score (*B* = −14.48, SE = 7.63, *t* = −1.90, *p* = 0.07, *R*^2^ = 0.166).

### Structural brain changes

#### Hippocampus and dorsolateral prefrontal cortex

**Figure 3:**
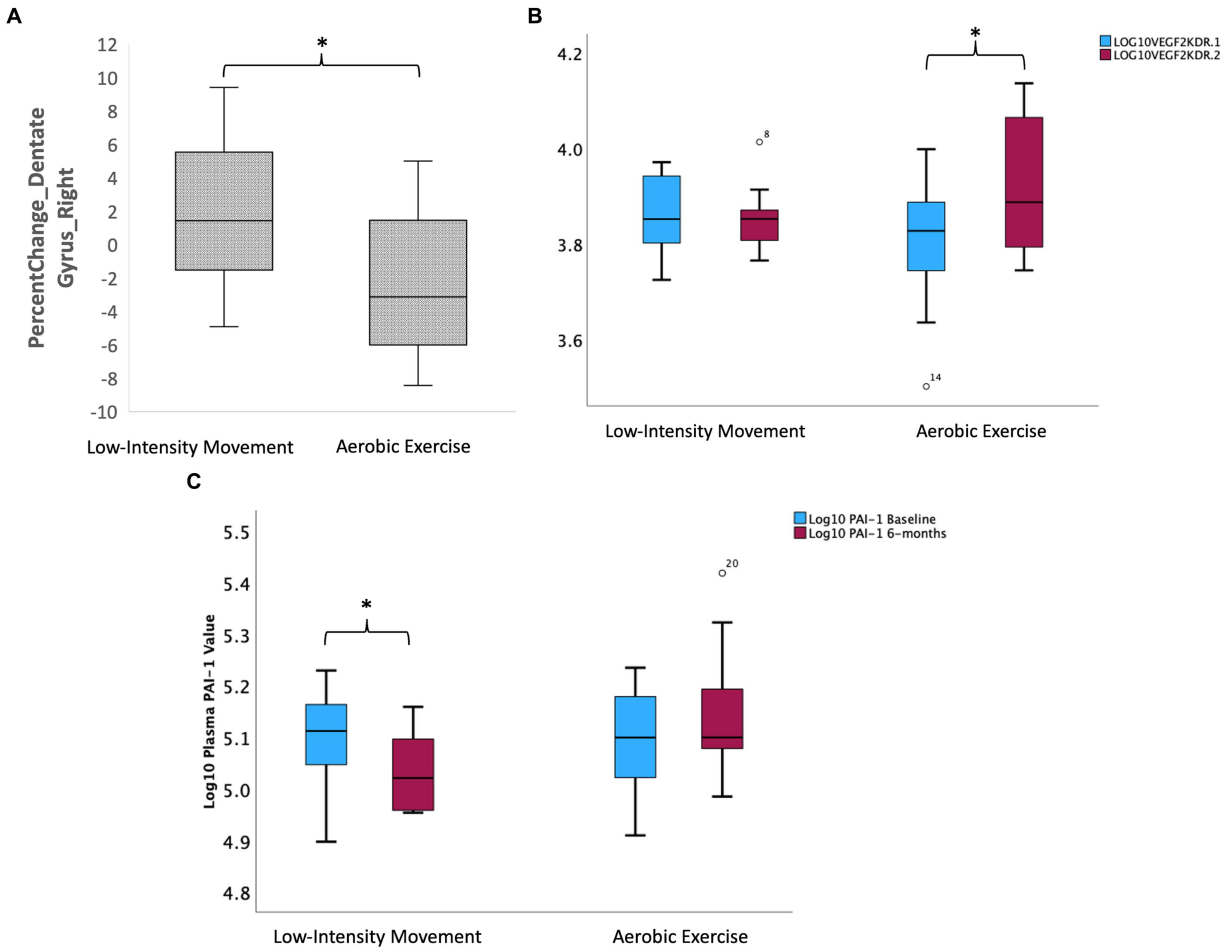
Intervention effects on hippocampal subfield volumes and peripheral inflammatory and neurotrophic factors. (A) Intervention effects on volume of the right dentate gyrus subregion of the hippocampus (LIM > AE). (B) Group differences in change in Log (VEGFR2KDR) over 6 months such that AE led to increase in VEGF2KDR but not LIM. (C) Group differences in change in PAI-1 over 6 months such that LIM led to decline in PAI-1 but not AE.

The AE and LIM groups showed differential percent change in right dentate gyrus volume over 6 months [LIM > AE (see Figure 3A); Independent samples t-test, (*t* = 2.13, df = 21, *p* = 0.045)]. No other hippocampal subfields showed differential change in volume over 6 months (all *p*s > 0.05). The LIM and AE groups also did not differ on percent change in dorsolateral prefrontal cortex volume, thickness, or surface area (all *p*s > 0.05).

#### Exploratory analyses: intervention effects on circulating inflammatory and neurotrophic factors

Intervention groups showed differential percent change in VEGF signaling over 6 months, such that only the intermittent AE group showed an increase in circulating VEGFR2KDR (Independent samples t-test *t* = −3.02, df = 22, *p* = 0.004; paired samples t-test in AE group *t* = −3.01 k df = 13, *p* = 0.01; see Figure 3B). The intermittent LIM group alone showed a decline in the pro-inflammatory PAI-1, which is implicated in both Alzheimer’s disease and memory decline [Independent samples t-test (*t* = 2.62, df = 22, *p* = 0.016); paired samples t-test in LIM group *t* = 3.82 df = 9, *p* = 0.004; See Figure 3C]. Intervention group differences for all other biomarkers tested (BDNF, IL-6, CRP) were not significant (*p* > 0.05) for all tests.

#### Possible peripheral and neuroimaging biomarkers of exercise-related cognitive improvement

**Figure 4:**
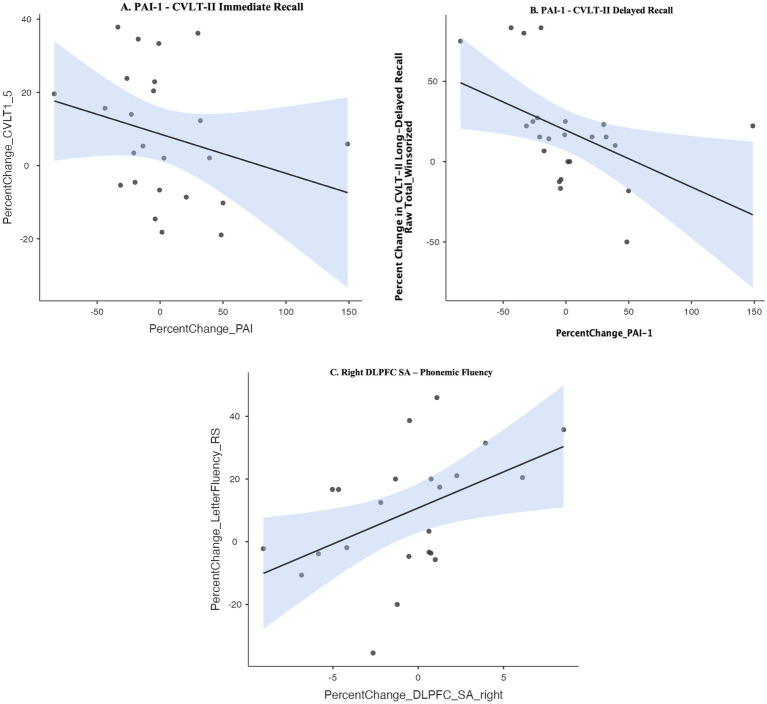
(A,B) Spearman’s rho correlation between improvement in learning and memory and decrease in peripheral levels of Plasminogen activator inhibitor-1 (PAI-1). (C) Spearman’s rho correlation between improvement in phonemic fluency and increase in right dorsolateral prefrontal cortex (DLPFC) surface area. CVLT-II, California Verbal Learning Test, 2nd edition.

We used non-parametric Spearman’s rho correlations to probe potential structural neuroimaging markers that relate to cognitive improvement in response to intermittent AE relative to LIM. Given the intermittent AE group showed selective improvement in executive functioning over 6 months (i.e., set-shifting, phonemic fluency), we examined whether improvement in executive functioning correlated with (1) morphological change in the DLPFC. We observed a correlation between improvement in phonemic fluency and increase in right DLPFC surface area (Spearman’s rho *r* = 0.56, *p* = 0.006) in the overall sample (see Figure 4C).

We further explored correlations between improvement in executive functioning measures (i.e., phonemic fluency, set-shifting) and change in serologic levels of neurotrophic and inflammatory factors previously shown to be involved in executive function (BDNF, VEGF, IL-6, CRP). Improvement in set-shifting or phonemic fluency was not related to change in BDNF, VEGF, IL-6, or CRP in the overall sample.

Given the LIM group showed differential improvement in learning and memory, we examined correlations between learning and memory improvement and morphological changes in neuroimaging markers consistently implicated in learning and memory (whole hippocampal volume, dentate gyrus hippocampal subfield volume). Improvement in learning and memory was not correlated with increase in volume of whole hippocampus or dentate gyrus (all p’s > 0.10), though the LIM group showed selective increase in right dentate gyrus volume (see Figure 3A). We further explored correlations between learning and memory improvement and change in circulating levels of inflammatory and neurotrophic factors previously implicated in learning and memory (BDNF, PAI-1). Improvement in learning and memory recall on the CVLT-II was correlated with a *decline* in in peripheral levels of PAI-1 (Spearman’s rho CVLT-II Learning: *r* = −0.41, *p* = 0.04; CVLT-II Short-Delayed Recall *r* = −0.57, *p* = 0.004; CVLT-II Long-Delayed Recall *r* = −0.57, *p* = 0.003) but not with change in peripheral BDNF (see Figures 4A,B). In linear regression models adjusting for WRAT-4 Reading Score, the association between percent change in PAI-1 and CVLT-II Long-Delayed Recall remained significant (*B* = −0.33, SE = 0.15, *t* = −2.23, *p* = 0.03, *R*^2^ = 0.23) but associations with percent change in CVLT-II Learning and Short-Delayed Recall became non-significant (CVLT short-delay recall: *p* = 0.09; CVLT learning: *p* = 0.26).

## Discussion

This pilot intervention study examined the cognitive and structural brain changes related to 6 months of intermittent AE vs. LIM. Consistent with our hypothesis and with the results of previous prolonged AE training interventions involving longer continuous AE bouts in older adults, our pilot trial replicated improvements in executive function (i.e., speeded set-shifting) in response to *intermittent* moderate-intensity AE, which was robust to baseline group differences in premorbid IQ (LIM > AE). Also consistent with previous research, we observed that improvement in executive functioning (i.e., phonemic fluency) correlated with an increase in DLPFC morphological integrity (Friedman and Robbins, 2022; Yuan and Raz, 2014).

Our results are inconsistent with a dose–response hypothesis of cognitive and structural brain changes of intermittent moderate-intensity AE vs. LIM, based on which we expected to observe similar but *greater* cognitive and brain changes in response to intermittent moderate-intensity AE relative to LIM. Rather, our results revealed *distinct* cognitive and regional structural brain changes with intermittent AE vs. LIM. Our results revealed an improvement in learning and memory performance in response to intermittent LIM but not in response to intermittent AE. Sensitivity analyses suggested that selective improvement in learning in response to LIM may in part be related to baseline group differences in estimated premorbid IQ (LIM > AE; WRAT-Reading score).

Our exploratory circulating biomarker analyses also unexpectedly revealed an attenuating effect of intermittent LIM (but not AE) on peripheral levels of the inflammatory marker PAI-1, which has previously been implicated in progression of memory decline in Alzheimer’s disease (Oh et al., 2014). Rodent studies have shown that amyloid beta plaques correlate with upregulation of PAI-1 and PAI-1 prevents appropriate cleavage of BDNF, leading to lower levels of mature BDNF, which is critical for cell proliferation, survival, and synaptic plasticity and cellular analogs of learning. As such, previous reports have suggested PAI-1 may be a key mechanism of inadequate neurotrophic support and subsequent neurodegeneration in Alzheimer’s disease (Gerenu et al., 2017).

Though there have been numerous exercise trials in community-dwelling older adults targeting cognitive aging, the key novelty of this trial design was the prescription of intermittent exercise, in short 10-min bouts multiple times per day. The health-related benefits of PA likely do not depend on bout duration, per the 2018 National Physical Activity Guidelines (Jakicic et al., 2019; Piercy et al., 2018); however, engaging in short bouts of exercise amounting to weekly recommended PA guidelines can achieve similar health benefits as fewer, longer PA bouts typically prescribed in exercise interventions (i.e., three weekly 50-min PA session) while the incremental nature of 10-min bouts of exercise can promote overall exercise intervention adherence (Jakicic et al., 1995).

The key implications of these pilot trial results were two-fold: (1) AE relative to LIM, even when accumulated in three prescribed intermittent 10-min bouts per day over 6 months, is sufficient to improve executive function, and (2) surprisingly, intermittent LIM relative to AE over 6 months may have valuable benefits for memory performance. Given activities similar to the LIM used in this study are frequently used as a control condition among exercise trials in cognitive aging, the cognitive benefits of the low-intensity PA conditions in trials are seldom highlighted in publications. We observed that intermittent LIM may be a feasible intervention in community-dwelling older adults with selective memory benefits. Further, exploratory analyses revealed a possible decrease in circulating PAI-1 correlated with LIM-related learning and memory improvement, suggesting PAI-1 may have potential of serving as a biomarker of the cognitive benefits of LIM. These results are meaningful, despite the pilot nature of this study, as learning and memory decline is the primary cognitive domain affected early in Alzheimer’s disease, the most common neurodegenerative condition. Interventions capable of improving learning and memory can delay time to dementia diagnosis and improve functional outcomes among those at high-risk for Alzheimer’s disease. These results are further notable because AE trials targeting cognitive aging consistently report gains in executive functioning yet infrequently report improvement in learning and memory.

Key limitations to this study include the small sample size due to the pilot nature of this trial; imbalanced intervention group sizes (1.5:1); a two-arm intervention design lacking a no-contact control group; limited generalizability of the results due to relatively homogenous demographic characteristics of the sample (mostly white women); and the inability to examine unique effects of intermittent prescribed exercise vs. traditional prolonged bouts of exercise within the same trial. However, key strengths of this study included the novelty of the intermittent exercise prescription among community-dwelling older adults, the multi-level characterization of possible biological process (i.e., structural neuroimaging markers of brain health and peripheral biomarkers related to neurotrophic support and inflammation) that may help explain exercise effects on cognition.

In conclusion, the results of this pilot study in community-dwelling older adults highlighted the feasibility value of 6 months of intermittent light-and moderate-intensity PA for cognition and brain aging, with possibly distinct cognitive and brain health benefits of intermittent AE vs. LIM that need to be replicated in larger, well-powered studies.

## Data Availability

The raw data supporting the conclusions of this article will be made available by the authors, without undue reservation.

## Footnotes

^1^ http://surfer.nmr.mgh.harvard.edu/
